# Plasma Levels of Tissue-Type Plasminogen Activator (tPA) in Normal Aging and Alzheimer's Disease: Links With Cognition, Brain Structure, Brain Function and Amyloid Burden

**DOI:** 10.3389/fnagi.2022.871214

**Published:** 2022-06-07

**Authors:** Clémence Tomadesso, Sara Martinez de Lizarrondo, Carine Ali, Brigitte Landeau, Florence Mézenge, Audrey Perrotin, Vincent de La Sayette, Denis Vivien, Gaël Chételat

**Affiliations:** ^1^Normandie Univ, UNICAEN, INSERM, UMR-S U1237, PHIND, Blood and Brain @ Caen Normandy Institute, Caen, France; ^2^Department of Clinical Research, CHU Caen-Normandie, Caen, France

**Keywords:** tissue plasminogen activator, biological markers, normal aging, Alzheimer's disease, FDG-PET, MRI, Flobetapir-PET, cognition

## Abstract

Tissue-type plasminogen activator (tPA) is a protease known for its fibrinolytic action but is also involved in physiological and pathophysiological aging processes; including amyloid elimination and synaptic plasticity. The aim of the study was to investigate the role of tPA in cognitive and brain aging. Therefore, we assessed the links between tPA plasma concentration and cognition, structural MRI, FDG-PET and Flobetapir-PET neuroimaging in 155 cognitively unimpaired adults (CUA, aged 20-85 years old) and 32 patients with Alzheimer's disease (ALZ). A positive correlation was found between tPA and age in CUA (*p* < 0.001), with males showing higher tPA than females (*p* = 0.05). No significant difference was found between ALZ patients and cognitively unimpaired elders (CUE). Plasma tPA in CUA negatively correlated with global brain volume. No correlation was found with brain FDG metabolism or amyloid deposition. Age-related tPA changes were associated to changes in blood pressure, glycemia and body mass index. Within the ALZ patients, tPA didn't correlate with any cognitive or neuroimaging measures, but only with physiological measures. Altogether our study suggests that increased tPA plasma concentration with age is related to neuronal alterations and cardiovascular risk factors.

## Highlights

- tPA plasma concentration (tPA PC) increases with age, but not with AD pathology.
- tPA PC is linked to cardiovascular health variables (arterial pressure and glucose) in aging.
- In aging there is a link between gray matter loss and tPA PC, not associated with cognitive decline.

## Introduction

Physiological aging is typified by brain molecular, cellular, structural, and functional changes, leading to a progressive cognitive decline. These age-related brain changes include decreases in gray matter volume and glucose metabolism, especially in frontal, cingulate and insular cortices, but also sensorimotor, and perisylvian regions and to a lesser extend parietal and temporal areas, including the hippocampus (Kalpouzos et al., 2009; Fjell and Walhovd, 2010; Chételat et al., 2013). Moreover, age is also associated with a significant increase in β-amyloid (Aβ) deposition measured with positron emission tomography (PET). The prevalence of amyloid pathology increasing from age 50 to 90 years from 10 to 44% in cognitively unimpaired individuals (Jansen et al., 2015). Collectively, these brain changes contribute to the decline in cognitive function, especially in executive functions and episodic memory (Fjell and Walhovd, 2010; Yuan and Raz, 2014). Regional decrease in gray matter volume and glucose metabolism and the presence of Aβ deposition are known to be associated with increased risk for Alzheimer's disease (AD) and actually are used as biomarkers in the diagnosis of the disease (McKhann et al., 2011; Dubois et al., 2014; Jack et al., 2018). The biological mechanisms underlying age and AD-related brain changes are still poorly understood and the identification of active or contributing factors is crucial for prevention and treatment.

Tissue-type Plasminogen Activator (tPA) is a protease synthesized and released by various cellular types such as endothelial cells, neurons, microglia, and oligodendrocytes (Louessard et al., 2016). tPA is essentially known for its fibrinolytic action by converting plasminogen into plasmin (Plow et al., 1995). In addition, tPA has other crucial functions in the brain, the control of memory and learning processes (Madani et al., 1999; Calabresi et al., 2000; Obiang et al., 2012), which has been particularly well documented in the classical model of memory, Long Term Potentiation (LTP; Qian et al., 1993; Huang et al., 1996; Baranes et al., 1998; Madani et al., 1999; Pang and Lu, 2004). tPA also modulates neuronal fate *via* several pathways, including neuroinflammation or excitotoxicity, and through proteolytic and non-proteolytic mechanisms (Plow and Hoover-Plow, 2004; Mehra et al., 2016). Moreover, tPA is involved in amyloid degradation processes through plasmin conversion (Kingston et al., 1995; Ledesma et al., 2000; Tucker et al., 2000; Melchor et al., 2003; Oh et al., 2014; Akhter et al., 2018). Interestingly, tPA plasma concentration was found to correlate with cardiovascular disease-related traits, such as triglycerides, total cholesterol, systolic blood pressure, diastolic blood pressure, body mass index, or fasting insulin level (Eliasson et al., 1994a,b, 1997; Koike et al., 1998; Lowe et al., 2004; Asselbergs et al., 2007).

Memory problems, inflammation, amyloid deposition, neurodegeneration and cardiovascular diseases are relevant factors associated with physiological and pathological aging and impact the quality of life and the risk for developing dementia. In this context, it seems particularly relevant to assess how tPA plasma levels are affected by normal aging and AD and how they relate to these relevant factors (memory problems, inflammation, amyloid deposition, neurodegeneration and cardiovascular diseases) in normal and pathological aging. A paper from Roussel et al. (2009) reported that whereas aging does not have an effect on the expression and proteolytic activity of tPA in the intravascular space, it had a significant impact on its expression and activity in the brain parenchyma. As for the effects of pathological aging on tPA plasma levels, previous studies reported discordant findings, with studies showing either decrease (see Whelan et al., 2019; Ziliotto et al., 2021 for review), increase (Hagnelius et al., 2010), or no differences (Marksteiner et al., 2014) in AD patients compared to controls .

In the present study, we measured plasma tPA levels in a large cohort of cognitively normal individuals encompassing the entire adult lifespan, as well as in patients with AD, to assess changes in tPA levels in non-pathological aging and AD. Moreover, our secondary objective was to assess the relationships between these potential changes in tPA plasma levels and biological cardiovascular markers, cognition, brain structure, glucose metabolism and amyloid load, as relevant markers of aging and AD.

## Methods

### Participants

One hundred and eighty-seven native French-speaking participants from the “Imagerie Multimodale de la maladie d'Alzheimer à un stade Précoce” (IMAP+) study (Caen) were included in the present study. The IMAP+ study was approved by a regional ethics committee (Comité de Protection des Personnes Nord-Ouest III) and registered with clinicaltrials.gov (no. NCT01638949). All participants gave their written informed consent to the study prior to the investigation. Three groups were used in the present study: (i) a group of 155 cognitively unimpaired adults (CUA) aged between 21-85 years old; (ii) a subsample of the CUA consisting in 60 cognitively unimpaired elders (CUE) aged between 60-85 years old; and (iii) a group of 32 cognitively impaired patients on the Alzheimer's continuum (ALZ) (aged between 56-85 years old)– i.e., with a positive florbetapir-PET scan using previously published methods (La Joie et al., 2012) including 14 patients with amnestic Mild Cognitive Impairment (aMCI) and 18 patients with dementia. Some of them have been included in previous publications from our laboratory (La Joie et al., 2014; Tomadesso et al., 2015, 2018). All participants were aged over 18 years, had at least 7 years of education, and had no history of alcoholism, drug abuse, head trauma, or psychiatric disorder.

Participants were recruited from the community and were classified as CUA if they performed within the normal range on all neuropsychological tests in a cognitive battery assessing multiple domains of cognition (verbal and visual episodic memory, semantic memory, language skills, executive functions, visuospatial functions and praxis). The majority of CUA underwent a florbetapir-PET scan, and the proportion of amyloid-positive scans was 20.0% (12/60) within the CUE and 12.2% (14/101) within the entire CUA group. ALZ patients (aMCI and patients with dementia) were recruited from local memory clinics and selected according to internationally agreed criteria. aMCI patients were selected based on Petersen's criteria for aMCI (Petersen and Morris, 2005) and patients with dementia fulfilled standard National Institute of Neurological and Communicative Disorders and Stroke, and Alzheimer's Disease and Related Disorders Association (NINCDS-ADRDA) clinical criteria for probable AD (McKhann et al., 1984). Clinical diagnosis was assigned by consensus under the supervision of a senior neurologist (VdLS) and two neuropsychologists (AP and SE). ALZ patients should meet both conditions of being classified as either aMCI or dementia and showing a positive florbetapir scan.

### Biological Data Measurements

Fasting blood samples were collected between 8 and noon to control for circadian variations. Then, multiple analyses were performed in the biological analyses center of Caen's hospital. Blood was collected into silicon-coated tubes of ethylenediaminetetraacetic acid (EDTA) for platelets and into fluorinated tubes for glucose analyses (glycemia). Platelet count was determined by XN-9000 IV CI Sysmex. Glycemia were determined by Beckman colorimetry DXC 800.

To measure plasmatic tPA antigen levels, venous blood samples, collected into silicon-coated tubes of trisodium citrate were processed to obtain platelet free plasma, thanks to 2 successive centrifugations (the first at 1500 g and +4C° for 15 mins and the second at 10000 g and 4 C° for 3 mins). Plasma aliquots were immediately transferred to plastic tubes, rapidly frozen, and stored at −80°C. Total tPA antigen (free and complexed to PAI-1) plasma concentration was measured by the Quantikine ELISA Human t-Plasminogen Activator/tPA Immunoassay (R&D) following manufacter instructions.

Systolic and Diastolic blood pressure measurements were performed before magnetic resonance imaging (MRI) and PET scans.

Weight as kilograms and height as meters of each participant were used to calculate Body Mass Index (BMI; defined as the body mass divided by the square of the body height, and is universally expressed in units of kg/m2, resulting from mass in kilograms and height in meters).

### Neuropsychological Data Assessment

Participants underwent an extensive neuropsychological evaluation as previously described (La Joie et al., 2016; Tomadesso et al., 2018). To obtain robust proxies of cognitive abilities, composite scores were created for executive functions, verbal abilities, and episodic memory (Supplementary Methods). For all composite scores, only scores showing no ceiling nor floor effects were used and higher values indicated better performances. Global cognition was measured using the Mini Mental State Examination (MMSE) and Mattis scale. Depressive symptomatology and anxiety (state and trait) were assessed using the Montgomery-Asberg Depression Rating Scale (Depression scale) and Spielberger-State Anxiety Inventory (STAI) (STAI-state, evaluated just after the blood sample), respectively.

### Imaging Data Acquisition

All participants underwent neuroimaging scans on the same MRI and PET scanners at the Cyceron Centre (Caen, France).

#### MRI Data

High-resolution T1-weighted anatomic images were acquired on a Philips Achieva 3T scanner (Philips, Eindhoven, the Netherlands) using a 3-dimensional fast-field echo sequence (sagittal; repetition time = 20 milliseconds, echo time = 4.6 milliseconds, flip angle 5 208, number of slices = 170, slice thickness = 1 mm, field of view = 256 × 256 mm^2^, matrix = 256 × 256).

#### PET Data

Both ^18^F-fluorodeoxyglucose (FDG)—and florbetapir-PET scans were acquired on a Discovery RX VCT 64 PET-CT device (GE Healthcare) with a resolution of 3.76 × 3.76 × 4.9 mm (field of view = 157 mm). Forty-seven planes were obtained with a voxel size of 2.7 × 2.7 × 3.27 mm. A transmission scan was performed for attenuation correction before the PET acquisition. For FDG-PET, participants, fasted for at least 6 hours before scanning, were at rest in a quiet and dark room before tracer injection. Approximately 180 MBq of FDG was injected intravenously 50 mins before a 10-min acquisition scan. For the amyloid-PET scan, ≈ 4 MBq/kg of florbetapir was injected intravenously 50 mins before a 20-min acquisition scan.

### Imaging Data Preprocessing

MRI data were segmented, normalized, and modulated for nonlinear warping with the Statistical Parametric Mapping 12 software (Wellcome Trust Centre for Neuroimaging, London, UK; http://www.fil.ion.ucl.ac.uk/spm/software/spm5/). Resulting local Gray Matter (GM) volume maps corrected for brain size were finally smoothed with a Gaussian kernel of 8 × 8 × 8 mm^3^ (x, y, z).

PET data were first corrected for partial volume effects (PMOD Technologies, Zurich, Switzerland). Resultant images (gray matter compartment only) were coregistered onto their corresponding MRI and normalized with the use of the deformation parameters derived from the MRI procedure. Images were then scaled with the cerebellar gray matter used as a reference to obtain standardized uptake value ratio images. Resulting PET images were finally smoothed with a Gaussian kernel of 10 × 10 × 10 mm^3^ (x, y, z). Each image from each processing step has been carefully checked visually for quality control by experienced raters.

First, for each CUA participant and ALZ patient, global values of gray matter volume, glucose metabolism and amyloid deposition were extracted from the resulting images for correlation analyses with tPA plasma concentration. Thus, global gray matter volume and glucose metabolism, were extracted from gray matter volume maps and FDG PET unsmoothed images respectively using a mask corresponding to the whole brain gray matter except the cerebellum. Global amyloid brain load was extracted from PET-florbetapir images using a mask corresponding to the entire gray matter except the cerebellum, occipital and sensory motor cortices, hippocampi, amygdala, and basal nuclei, as described previously (La Joie et al., 2012).

Second, the resulting images (after smoothing) were also used for voxelwise multiple regression analyses with tPA plasma concentration (see below).

### Statistical Analyses

We first aimed at assessing whether ALZ and physiological aging impacted tPA plasma concentration. The corresponding analyses are described below in sections Group Comparisons and Effect of Age on tPA Plasma Concentration respectively. Second, we aimed at assessing whether these changes were related to changes in cognition or brain structure, glucose metabolism and amyloid load. The corresponding analyses are described in the sections Relationships Between tPA Plasma Concentration and Neuropsychological, Biological and Global Neuroimaging Measures and Voxelwise Neuroimaging Analyses.

#### Group Comparisons

A two sample t-test was performed to compare each continuous data (age, education level, MMSE, tPA plasma concentrations) between the CUE and ALZ patients. Categorical variables (sex and apolipoprotein E (APOE) genotype) were compared using proportional χ^2^ tests (Pearson) (see Table 1). Outlier tPA plasma concentration values were detected using Tukey two-sided test with a coefficient of 1.5 and repeated iteratively until all outliers have been identified. One outlier was detected; all analyses with tPA were performed after excluding this outlier (*n* = 1). All results were considered significant at *p* < 0.05.

**Table 1:** Demographic, clinical and tPA plasma concentration data.

**Measure**	**CUA (*n* = 154)**	**CUE+ (*n* = 59)**	**ALZ patients (*n* = 32)**	**CUE vs. ALZ patients (*p* value)**
Age year	51.2 ± 18.9	70.7 ± 6.5	72.0 ± 8.5	0.4
Sex % Male	46.8	42.4	65.6	0.03
Education year	13.7 ± 7.8	12.6 ± 3.9	11.6 ± 3.4	0.2
MMSE total score, /30	29.2 ± 1.0	28.8 ± 1.1	23.6 ± 4.5	<0.001
APOE ε4 carrier % carriers	26.6	25	70	<0.001
tPA pg/mL	3,844.3 ± 1,556.2	4,670.3 ± 1,430.3	4,461.9 ±1,530.6	0.5

*Between-group differences were assessed using t-tests for continuous variable and X^2^ test for categorical variable. MMSE, Mini Mental State Examination; tPA, tissue Plasminogen Activator; CUA, Cognitively Unimpaired Adults; CUE, Cognitively Unimpaired Elders. +: CUE cases are a sub-group of the CUA*.

#### Effect of Age on tPA Plasma Concentration

A correlation analysis between age and tPA level within the entire CUA group was performed to test for an effect of physiological aging on tPA plasma concentration. For the sake of completeness, we also tested for an effect of sex and education on tPA plasma levels.

#### Relationships Between tPA Plasma Concentration and Neuropsychological, Biological and Global Neuroimaging Measures

Within each group, tPA concentration was entered as a dependent variable in separate partial correlation models with each neuropsychological, biological and neuroimaging data (global amyloid load, global gray matter volume and global brain glucose metabolism) as an independent variable, correcting for age and sex. The same analyses were then performed without correction for age and sex.

#### Voxelwise Neuroimaging Analyses

When a significant link was found with global neuroimaging measures, then voxelwise multiple regression analyses were performed between tPA plasma concentration and the corresponding neuroimaging modality, with age and sex as a nuisance variable. To address the issue of multiple comparisons, for each imaging modality a cluster size was determined combined with a voxel-level *p* (uncorrected) <0.005 threshold to achieve a corrected statistical significance for multiple comparisons of *p* < 0.05 (as determined through Monte Carlo simulations using the AlphaSim program).

## Results

### Group Comparisons

**Figure 1:**
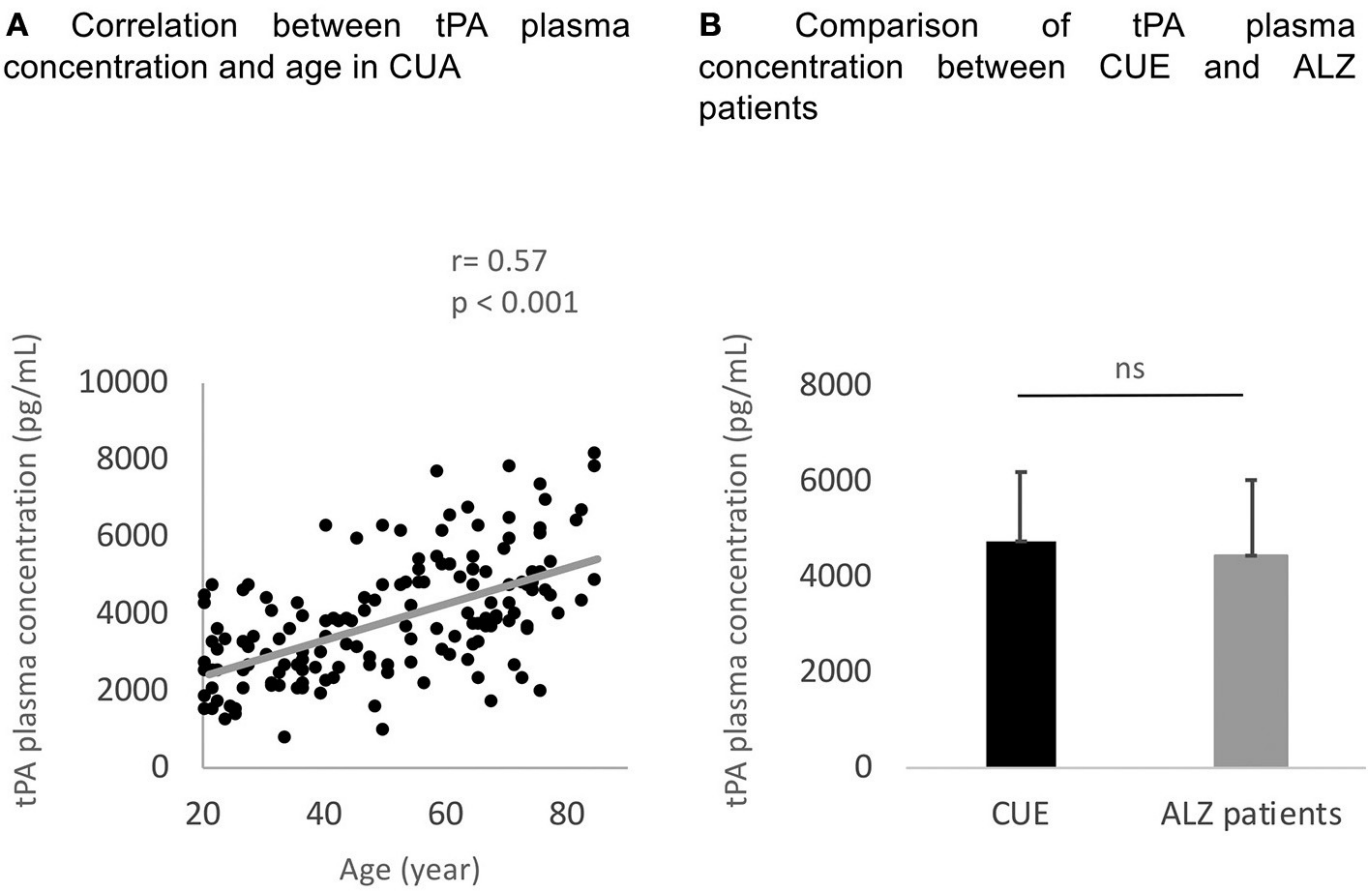
Correlation between age and tPA concentration within the CUA **(A)** and comparison of tPA plasma concentration between CUE and ALZ patients groups **(B)**. tPA, tissue Plasminogen Activator; CUA, Cognitively Unimpaired Adults; ns, no statistically significant difference.

The results of the group comparisons are reported in Table 1. ALZ patients did not differ from CUE in age and education level, whereas ALZ patients had a higher proportion of male in comparison to CUE. As expected, the proportion of APOE ε4 carriers and the global amyloid load value were higher, and the mean MMSE score was lower, in the ALZ patients compared to the CUE. No difference in tPA concentration was found between ALZ patients and CUE (Figure 1).

### Effect of Age on tPA Plasma Concentration

As shown in Figure 1, tPA plasma concentration significantly correlated to age (R = 0.56 and *p* < 0.001). Males tended to show higher tPA plasma concentration than females (t = 1.96 and *p* = 0.05). Education level was not correlated to tPA plasma concentrations (R = −0.11 and *p* = 0.17).

### Relationships Between tPA Plasma Concentration and Neuropsychological, Biological and Global Neuroimaging Measures

Within the group of CUA, negative correlations were found between tPA plasma concentration and global cognition, executive functions and episodic memory composite scores; these correlations were no longer significant when correcting for age and sex (Table 2). Regarding biological measures, tPA plasma concentrations correlated positively with systolic, diastolic blood pressure, glycemia and BMI, and the same results were found when correcting for age and sex (although as a trend for systolic blood pressure; see Table 2). Finally, a significant correlation was found between tPA concentration and global gray matter volume and glucose metabolism; only the relationship with global gray matter volume remained significant when correcting for age and sex (Table 2). No significant relationship was found with global amyloid load.

**Table 2:** Results of the correlation analyses between tPA plasma concentration and neuropsychological, biological as well as neuroimaging measures in CUA (*n* = 154).

	**Mean ±SD**	***n***	**Correlations in CUA**			
			**R Uncorrected**	***p* Uncorrected**	**R Correcting for age and sex**	***p* Correcting for age and sex**
**Neuropsychological measures**:						
MMSE total score, /30	29.2 ± 1.0	150	−0.20	**0.02**	−0.05	0.54
Mattis total score, /144	141.8 ± 2.5	152	−0.10	0.21	−0.03	0.75
STAI state score, /80	26.5 ± 6.5	148	0.004	0.96	−0.02	0.82
STAI trait score, /80	36.7 ± 8.9	152	0.03	0.73	0.03	0.74
Depression scale Score, /60	1.3 ± 2.3	148	−0.03	0.72	0.04	0.66
Verbal abilities composite score	−0.02 ± 0.8	83	0.06	0.61	0.05	0.69
Executive function composite score	0.01 ± 0.7	149	−0.35	***p*** **<** **0.001**	−0.09	0.27
Episodic memory composite score	0.002 ± 0.7	148	−0.34	***p*** **<** **0.001**	−0.11	0.20
**Biological measures**:						
Systolic blood pressure (mmHg^−1^)	130.7 ± 21.0	151	0.37	***p*** **<** **0.001**	0.15	0.07
Diastolic blood pressure (mmHg^−1^)	76.6 ± 11.2	151	0.36	***p*** **<** **0.001**	0.23	**0.004**
Platelets concentration (g/L)	247.7 ± 57.0	146	−0.14	0.09	−0.09	0.29
Glycemia (mmol/L)	5.24 ± 0.7	147	0.45	***p*** **<** **0.001**	0.23	**0.006**
BMI (Kg/m^2^)	24.5 ± 2.9	68	0.43	***p*** **<** **0.001**	0.41	***p*** **<** **0.001**
**Neuroimaging measures**:						
Global gray matter volume	0.7 ± 0.06	152	−0.57	***p*** **<** **0.001**	−0.17	**0.04**
Global glucose metabolism	1.1 ± 0.1	144	−0.38	***p*** **<** **0.001**	−0.02	0.84
Global amyloid load	1.0 ± 0.1	114	0.17	0.07	0.04	0.68

*Mean (SD) shown for each variable. R and p uncorrected correspond to correlation analyses, and, R and p corrected correspond to partial correlation correcting for age and sex. MMSE, Mini Mental State Examination; tPA, tissue Plasminogen Activator; CUA, Cognitively Unimpaired Adults; STAI, Spielberger-State Anxiety Inventory. Bold values are significant correlations (p <0.05)*.

Within the group of ALZ, there were no significant correlations between tPA plasma concentration and global cognition, executive functions and episodic memory composite scores; nor with global gray matter volume, glucose metabolism or global amyloid load; but significant correlations to glycemia and BMI (Table 3).

**Table 3:** Results of the correlation analyses between tPA plasma concentration and neuropsychological, biological as well as neuroimaging measures in ALZ patients (*n* = 32).

	**Mean ±SD**	***n***	**Correlations in ALZ patients**			
			**R Uncorrected**	***p* Uncorrected**	**R Correcting for age and sex**	***p* Correcting for age and sex**
**Neuropsychological measures**:						
MMSE total score, /30	23.6 ± 4.5	30	0.28	0.14	0.22	0.25
Mattis total score, /144	124.3 ± 12.6	30	0.12	0 0.52	0.10	0.60
STAI state score, /80	34.3 ± 9.8	28	−0.20	0.31	−0.004	0.98
STAI trait score, /80	39.3 ± 7.8	29	−0.18	0.35	−0.04	0.84
Depression scale score, /60	3.4 ± 3.2	30	−0.23	0.21	0.18	0.38
Verbal abilities composite score	−1.20 ± 1.1	28	0.30	0.12	0.05	0.69
Executive function composite score	−2.87 ± 3.2	23	0.09	0.70	0.19	0.41
Episodic memory composite score	−1.89 ± 0.5	19	−0.19	0.45	0.04	0.87
**Biological measures**:						
Systolic Blood Pressure (mmHg^−1^)	148.7 ± 25.9	28	0.37	0.05	0.32	0.11
Diastolic Blood Pressure (mmHg^−1^)	81.3 ± 12.3	28	0.15	0.45	0.05	0.80
Platelets concentration (g/L)	224.8 ± 41.7	20	−0.26	0.26	−0.18	0.47
Glycemia (mmol/L)	5.75 ± 0.9	25	0.58	**0.003**	0.23	**0.007**
BMI (Kg/m^2^)	25.4 ± 2.7	23	0.41	***p*** **<** **0.001**	0.39	***p** **<** **0.001***
**Neuroimaging measures**:						
Global gray matter volume	0.60 ± 0.06	32	−0.08	0.67	−0.29	0.13
Global glucose metabolism	0.98 ± 0.1	31	−0.02	0.1	0.62	0.31
Global amyloid load	1.6 ± 0.1	30	0.22	0.25	0.12	0.53

*Mean (SD) shown for each variable. R and p uncorrected correspond to correlation analyses, and, R and p corrected correspond to partial correlation correcting for age and sex. MMSE, Mini Mental State Examination; tPA, tissue Plasminogen Activator; STAI, Spielberger-State Anxiety Inventory. Bold values are significant correlations (p <0.05)*.

### Voxelwise Neuroimaging Analyses

**Figure 2:**
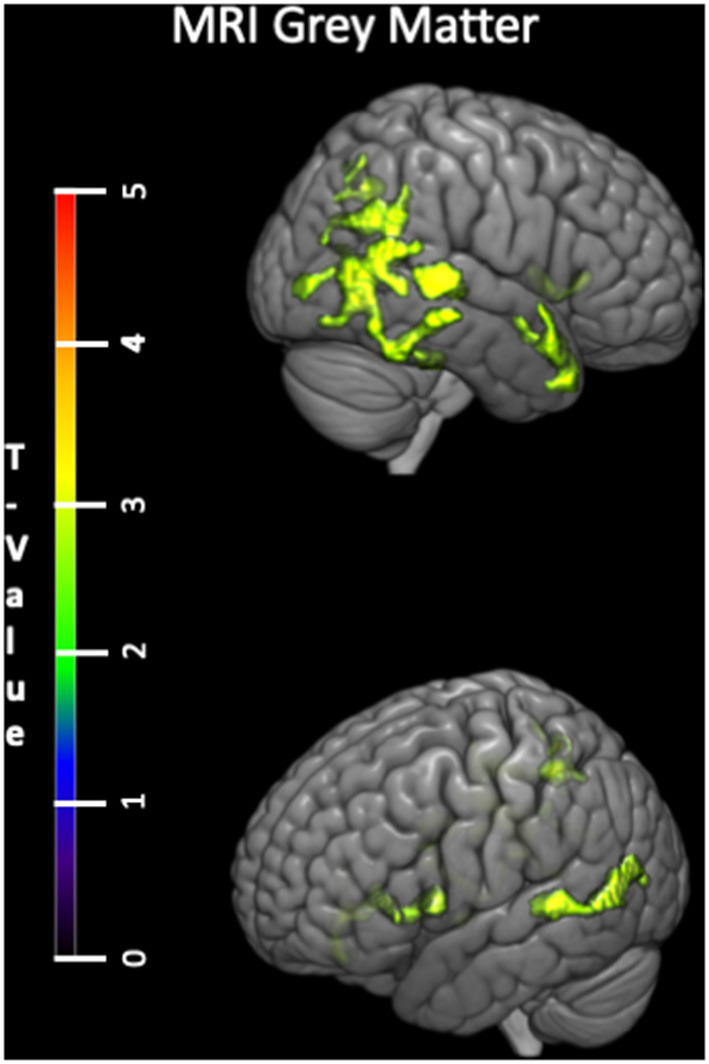
Voxelwise correlations between tPA plasma concentration and gray matter volume (negative correlation) within the CUA group with age and sex as covariates. tPA, tissue Plasminogen Activator; CUA, Cognitively Unimpaired Adults; MRI, Magnetic Resonance Imaging; FDG, 18F-fluorodeoxyglucose; PET, Positron Emission Tomography.

Voxelwise analyses within the CUA revealed that the negative correlation between tPA plasma concentration and gray matter volume was rather diffuse, involving several brain regions including the insula and medial orbitofrontal gyrus, temporal lateral regions and a region encompassing the precuneus, angular and supramarginal gyrus (Figure 2).

## Discussion

Our findings revealed that tPA plasma concentration increased with age while it was not affected in ALZ patients compared to CUE. Increase in tPA plasma concentration with age is consistent with a previous study showing similar effects on a smaller sample with lower age range (Rånby et al., 1986; Hashimoto et al., 1987). This increase might be a consequence of the fact that the hemostatic balance leans with aging toward thrombosis (Sepúlveda et al., 2015). Thus, cardiovascular events such as atherosclerosis and thrombosis increase in the elderly (Ridker et al., 1993), and tPA activity is known to increase in these conditions (Ridker et al., 1993, 2004). This interpretation is supported by positive correlations between tPA levels and glycemia, BMI, systolic and diastolic blood pressure—all related to cardiovascular health (Ridker et al., 1993, 2004; Stamler et al., 1993; Eliasson et al., 1994a,b, 1997; Koike et al., 1998; Coutinho et al., 1999). This is in line with the fact that tPA and age are both associated with cardiovascular diseases (Ridker et al., 1993, 2004; Eliasson et al., 1997; North and Sinclair, 2012) and that higher tPA antigen levels are predictive of future cardiovascular disease (Thögersen et al., 1998). This link between cardiovascular health measures and tPA plasma concentration was also partially highlighted in ALZ patients likely reflecting the fact that aging physiological processes are still ongoing concomitantly to AD pathology.

We found no difference between ALZ patients and CUE. It is possible however that AD is associated with changes in other plasma components that interact with tPA—such as plasminogen—thereby affecting tPA activity. Further studies are needed to investigate this question.

In our CUA sample, we also found a trend for a sex effect with higher tPA plasma concentration in males than females. This gender difference has been previously reported in similar populations (Takada and Takada, 1989; Gebara et al., 1995; Asselbergs et al., 2007) and could be explained by differences in sex hormones (Gebara et al., 1995). Indeed, high estrogen levels (only present in females) were found to be associated with lower levels of tPA antigen (Gebara et al., 1995; Segura et al., 2006). In opposition, testosterone was shown to be related to increased levels of tPA (Jin et al., 2007). This might reflect the higher prevalence of cardiovascular diseases in men than women (Regitz-Zagrosek et al., 2016; Hvas and Favaloro, 2017).

In the CUA, we found plasma tPA levels to be negatively associated with gray matter volume both as a global measure and voxelwise. These effects were rather diffuse and included both age-sensitive (insular and frontal cortex) and AD-sensitive (posterior temporal and parietal cortex) brain regions. It might reflect the role of tPA in excitotoxin-related neuronal degeneration (Tsirka et al., 1995; Nicole et al., 2001; Lee et al., 2015), or with brain vascular dysfunction and inflammation processes leading to gray matter atrophy (Breteler et al., 1994; Fein et al., 2000; Mayda et al., 2011; Papma et al., 2012; Iadecola, 2013). Note that no such relationship between tPA levels and global gray matter volume was found within the ALZ patients likely because AD-pathological processes take over tPA to lead gray matter loss in the patients.

No association was found between plasma tPA levels and brain glucose metabolism nor amyloid deposition in this study (neither in CUA nor in ALZ groups). Given the role of tPA in amyloid plaque degradation shown *in vitro* (Kingston et al., 1995; Ledesma et al., 2000; Tucker et al., 2000; Melchor et al., 2003; Oh et al., 2014; Akhter et al., 2018), the lack of correlation between plasma tPA level and brain amyloid load might appear surprising. This might be explained by a lack of relationships between plasmatic tPA and brain amyloid in AD patients, the gap between plasma tPA level and brain tPA activity (see also below), and/or the lack of statistical power associated with the low proportion of CUA with significant amount of amyloid in their brain (i.e. 12.2 %). Further studies on larger samples of amyloid-positive elderly would allow to further assess this question.

Importantly, the modifications in tPA levels and related impact in gray matter volume did not translate to age-independent decrease in cognitive function. Indeed, the relationships between increased tPA levels and decreased executive functions and episodic memory scores were not significant when corrected for age and sex. We can suppose that tPA-related brain changes are too diffuse, and tests are not sensitive enough, to measure a significant impact on cognition. tPA was found to be associated to memory and learning processes in animals (Madani et al., 1999; Calabresi et al., 2000; Obiang et al., 2012) but also in cellular and molecular models of memory (i.e. LTP) (Qian et al., 1993; Huang et al., 1996; Baranes et al., 1998; Madani et al., 1999; Pang and Lu, 2004). No previous study assessed the relationship between tPA and cognition in Human and future studies might further explore this question.

Another unresolved issue relates to the association between tPA plasma concentration and tPA brain concentration. *In vitro*, it has been demonstrated that tPA crosses the intact blood-brain barrier by low-density lipoprotein (LDL) receptor-related protein (LRP)-dependent transcytosis (Benchenane et al., 2005). Surprisingly, tPA concentration in the brain was found to be negatively correlated with age in mice whereas no differences were observed on in the proteolytic activity in the vascular compartment (Roussel et al., 2009). It is possible that the impact of multifactorial physiological aging on tPA levels observed in our study on human cannot been translated to mice. It could also be explained by an indirect relationship between plasma and brain tPA levels. Future studies are needed to shed light on this question.

The strength of the present study is to provide a comprehensive assessment of plasma tPA level changes with age and AD and their links with cognition and complementary measures of brain integrity thanks to multimodal neuroimaging. Another strength is that ALZ patients were selected not only based on clinical criteria (MCI or dementia) but also on the presence of brain amyloid deposition, which is known to increase the likelihood of AD etiology in MCI and dementia patients (Dubois et al., 2014; Jack et al., 2018). On the other hand, because the presence of amyloid deposition in cognitively normal elderly is frequent and associated with unknown clinical relevance (Chételat et al., 2020), CUA and CUE were selected irrespective of their amyloid status. Therefore, some of them (and more particularly some of the CUE (20%) were amyloid positive, which could partly reflect the fact that they are at a pre-clinical stage of AD. This study showed a strong linear increase in tPA from 20 to 80 years in CUA, mainly associated with diffuse gray matter volume loss, while no change was found in AD patients compared to age-matched controls. It opens avenues for future studies to explore the possible role of neurovascular and neuroinflammation processes, the relationships between plasma and brain tPA levels and total and active tPA levels, and the links between tPA and amyloid deposition in larger amyloid-positive samples. Finally, this study also has limitations inherent to cross-sectional studies and future studies might help further understand the role of tPA in age and AD-related brain and cognitive changes using a longitudinal design.

## Data Availability Statement

The original contributions presented in the study are included in the article/Supplementary Material, further inquiries can be directed to the corresponding author/s.

## Ethics Statement

The studies involving human participants were reviewed and approved by Comite de Protection des Personnes Nord Ouest Centre Hospitalier Universitaire Niveau 3—porte 508 Avenue de la Côte de Nacre CS 30001 14033 CAEN cedex 9. The patients/participants provided their written informed consent to participate in this study.

## Author Contributions

CT, SL, CA, AP, DV, and GC conceived and designed the study. CT, SL, BL, FM, AP, VL, and GC collected the data. CT, BL, and FM analysed the data. CT, SL, CA, AP, DV, and GC interpreted the data. CT, SL, and GC wrote the article. All authors critically reviewed the article. All authors contributed to the article and approved the submitted version.

## Funding

The study was supported by Fondation Plan Alzheimer (Alzheimer Plan 2008–2012), Programme Hospitalier de Recherche Clinique (PHRCN 2011-A01493-38 and PHRCN 2012 12-006-0347), Agence Nationale de la Recherche (LONGVIE 2007), Région Basse-Normandie, and Association France Alzheimer et maladies apparentées AAP 2013. Funding sources were not involved in the study design, data acquisition, data analysis, or manuscript writing.

## Conflict of Interest

AP currently works for Life Molecular Imaging GmbH, Berlin, Germany. The remaining authors declare that the research was conducted in the absence of any commercial or financial relationships that could be construed as a potential conflict of interest.

## Publisher's Note

All claims expressed in this article are solely those of the authors and do not necessarily represent those of their affiliated organizations, or those of the publisher, the editors and the reviewers. Any product that may be evaluated in this article, or claim that may be made by its manufacturer, is not guaranteed or endorsed by the publisher.
