# Supplementary material for: Plasma Levels of Tissue-Type Plasminogen Activator (tPA) in Normal Aging and Alzheimer's Disease: Links With Cognition, Brain Structure, Brain Function and Amyloid Burden
Source: Front Aging Neurosci. 2022 Jun 7;14:871214. doi: 10.3389/fnagi.2022.871214 (PMC9211060; doi:10.3389/fnagi.2022.871214)
Supplement: Supplementary file 1 [file Data_Sheet_1.PDF]

Supplementary Material

Supplementary Methods

Neuropsychological assessment

To obtain more robust proxies of cognitive abilities and minimize the issue of multiple statistical testing, composite cognitive scores were used instead of multiple (sub)tests. For that purpose, performances from different tasks that showed neither ceiling nor floor effects were z-transformed and averaged as follows:

- **Verbal abilities**
  - the semantic verbal fluency (number of animals in 2 min)
  - the number of correct responses in the Mill Hill Vocabulary test

- **Executive function**
  - TMT test (time difference between TMT part B and part A)*
  - Stroop test (time difference between the interference and color cards)*
  - the phonemic verbal fluency (number of words beginning with ‘‘p’’ in 2 min)
  - the digit span backwards

- **Episodic memory**
  - 2 free recalls from the *Encoding Storage Retrieval* (ESR) paradigm (two 16-word lists, one being encoded incidentally and superficially, the other after deep and intentional encoding)
  - 2 free recalls from a visual version of the ESR paradigm (based on two lists of nonfigurative graphical signs)

* note that before averaging, z scores derived from reaction times were reversed so that increasing values always indicated better performances)

Additional information on the original tests (ESR paradigm and its visual version) can be found in previous references from our lab:

Eustache F, Laisney M, Lalevée C, Pèlerin A, Perrotin A, Egret S, et al. Une nouvelle épreuve de mémoire épisodique : l’épreuve ESR-forme réduite (ESR-r), adaptée du paradigme ESR (encodage, stockage, récupération). Rev Neuropsychol 2015;me 7:217–25.

La Joie R, Landeau B, Perrotin A, Bejanin A, Egret S, Pélerin A, et al. Intrinsic connectivity identifies the hippocampus as a main crossroad between Alzheimer’s and semantic dementia-targeted networks. Neuron 2014;81:1417–28. doi:10.1016/j.neuron.2014.01.026.

Mevel K, Landeau B, Fouquet M, La Joie R, Villain N, Mézenge F, et al. Age effect on the default mode network, inner thoughts, and cognitive abilities. Neurobiol Aging 2013;34:1292–301. doi:10.1016/j.neurobiolaging.2012.08
